# Perspectives of Mining Personnel on Adopting Occupational Exoskeletons: Comparisons Between a Developed and a Developing Country

**DOI:** 10.1007/s42461-025-01189-1

**Published:** 2025-03-01

**Authors:** Feyisayo Akinwande, Sunwook Kim, Aanuoluwapo Ojelade, Khoirul Muslim, Hardianto Iridiastadi, Mahiyar Nasarwanji, Jeong Ho Kim, Maury A. Nussbaum

**Affiliations:** 1 Department of Industrial and Systems Engineering, Virginia Tech, Blacksburg, VA USA; 2 Faculty of Industrial Technology, Institut Teknologi Bandung, Bandung, Indonesia; 3 Pittsburgh Mining Research Division, National Institute for Occupational Safety and Health, Pittsburgh, PA USA; 4 Department of Environmental and Occupational Health, Texas A&M University, College Station, Texas, TX USA; 5 Department of Industrial and Systems Engineering, University at Buffalo, Buffalo, NY USA

**Keywords:** Work-related musculoskeletal disorders, Mining industry, Intervention, Technology adoption, Exoskeleton

## Abstract

Occupational exoskeletons (EXOs) have received growing attention as a new ergonomic intervention to reduce physical demands in various industries (e.g., manufacturing, logistics, construction, and agriculture). However, their potential use in mining has not yet been reported. Survey data (*n* = 135) were obtained from mining workers in the United States (US) and Indonesia (ID). Qualitative and frequency analyses were used to summarize and compare respondents’ perceived barriers, benefits, and promoters to EXO use and adoption. Beta regression analyses were also used to examine whether the perceived likelihood to use arm-support EXOs or back-support EXOs differed between the countries and was affected by demographic or job characteristics, or by perceptions regarding EXOs. Both US and ID respondents reported potential benefits of EXOs for physically demanding tasks such as lifting and overhead work, and they shared concerns about adaptation, uncertainty or lack of knowledge, confined spaces, device weight, potential failure or damage, and costs. However, some key differences also emerged: US respondents were more likely to consider using arm-support EXOs and back-support EXOs, despite expressing concerns about their use; ID respondents, although they reported more existing health and safety hazards, appeared more hesitant about adopting EXOs, possibly due to these additional hazards. These results demonstrate that miners appear to have an interest in EXOs but also emphasize the need to ensure task compatibility, comfort, and affordability to ensure the safe and effective adoption of EXO technology in mining in both developed and developing countries.

**Supplementary Information:**

The online version contains supplementary material available at 10.1007/s42461-025-01189-1.

## Introduction

Work-related musculoskeletal disorders (WMSDs) injuries or dysfunctions affecting muscles, bones, nerves, tendons, ligaments, joints, cartilage, and spinal discs [1] are a common problem and concern worldwide [2]. Mining workers continue to experience high rates of WMSDs. In the US, for example, a decade ago miners had an incidence rate of WMSDs of 46.4 per 10,000 full-time employees, higher than the overall industry rate of 31.9 [3]. More recently, in 2020, the incidence rate of WMSDs in mining was 31 per 10,000 full-time employees, which was also higher than the overall industry rate of 25.4 [4]. The back and shoulder are the two body regions most impacted (e.g., [5, 6]), accounting for 15.3% and 12.5% of all WMSD cases, respectively, and with medians of 30 and 99 lost workdays [4]. Many mining jobs involve frequent exposures to WMSD risk factors, such as handling bulky objects, forceful exertions, abrupt movements, and/or operating in confined spaces or non-neutral postures [7, 8].

WMSD risks and burden vary across countries. Industrially developing countries may face greater challenges due to differences in the type and volume of mined commodities, working conditions, and work cultures. For example, Indonesia, a leading coal exporter, has miners who experience a higher prevalence rate of WMSDs compared to those in developed countries like the US, UK, or Australia [9]. Specifically, Rabiei et al. [10] reported prevalence rates of 79% for neck WMSDs and 77% for upper back WMSDs among Indonesian miners, compared to respective values of 10% and 47% among US miners. Furthermore, Widanarko et al. [11] found that low back WMSDs were 23.7% more prevalent among Indonesian coal miners than in developed countries. These WMSDs, regardless of the country, have a substantial financial impact on stakeholders. In the US, for example, direct costs for workers’ compensations were estimated to be $20 billion, whereas indirect costs can be five times higher [12]. Therefore, effective interventions are needed to control WMSD risks in mining.

Engineering and administrative controls are common ergonomic interventions for preventing or controlling WMSDs. Engineering controls often require the redesign of jobs including factors such as equipment, tools, environments, and facilities. Administrative controls, in contrast, are management-led work practices and policies that are used to reduce or prevent exposures to WMSD risk factors [13]. Diverse controls have been implemented in mining, including redesign of seats and suspension systems for heavy mining vehicles [14–17]; equipment and tool design/redesign [18–22]; participatory ergonomics programs [23–25]; behavior-based safety programs [26]; and training programs [27–30]. Despite the reported efficacy and effectiveness of some of these approaches, there are often associated challenges such as high costs and limited feasibility in certain work settings [31]. Given the persistent high prevalence of WMSDs in mining, there remains a need for alternative, innovative interventions to address WMSDs across different regions, while accounting for potential region-specific challenges.

One such intervention is occupational exoskeletons (EXOs), wearable devices designed to assist/support the wearer during various physical activities, which are a promising emerging technology [e.g., 32–34]. Specifically, arm-support exoskeletons (ASEs) are designed to alleviate the loads on shoulder structures. These devices provide varying levels of torque, depending on shoulder elevation angle, to help elevate the arm. Back-support exoskeletons (BSEs) support the lower back and hips by generating external extension torques to counteract the effects of gravity and inertia on the upper body and handled loads. These two are the most common types of occupational EXOs at present (see exoskeletonreport.com), and most commercial ASEs and BSEs generate assistive torques through passive mechanisms (e.g., from elastic materials).

Existing evidence indicates that short-term use of EXOs can reduce physical demands and potentially improve task performance during various tasks, such as overhead work [35–37], static trunk bending [38], and repetitive lifting [39, 40]. Such benefits, however, are clearly contingent upon the specific EXO design and work task characteristics [41–44]. EXO use may also have adverse effects, such as on task performance [45], balance recovery ability [46–48], and wearer discomfort [38, 49]. These findings suggest the need for careful consideration when implementing EXOs in a specific workplace, considering both potential benefits and limitations.

EXO implementation has already been examined in several occupational sectors, including manufacturing [50–54], construction [55–57], and agriculture [58–61]. These studies have identified potential industry-specific tasks that may benefit most from EXO use, and such studies have helped identify promoters and barriers to EXO implementation and acceptance in these industries. Key factors for the latter include awareness, managing thermal discomfort, and heat-related illnesses, as well as addressing usability challenges within a given industry [62–64]. The unique demands of mining further suggest that successful implementation will require careful consideration of relevant work conditions and miner perceptions. To date, though, there are no reports (to our knowledge) on the use of EXOs in the mining industry.

We thus completed an exploratory study with two objectives. The first was to compare the perspectives regarding the facilitators of and barriers to EXO adoption among mining personnel in the US and Indonesia, representing a developed and developing country, respectively. The second objective was to examine how the perceived likelihood to use an EXO differed between these two countries. Findings from this study were intended to help guide future practices for adopting and implementing EXOs in the mining industry.

## Materials and Methods

### Participants and Study Design

The study involved an online survey, and the protocol was approved by the Virginia Tech Institutional Review Board. Involvement was entirely voluntary, and all participants gave informed consent prior to any data collection. To be eligible for the study, potential participants were required to have current or previous experience in mining operations; however, prior experience with EXOs was not required. Participants were recruited through our existing industry contacts, word-of-mouth referrals, and social media advertisements. The focus of these recruitment efforts was primarily on the US and Indonesia (ID). Upon completing the online survey, each participant was compensated with a $50 e-gift card. All responses to the survey were collected between August and November of 2022.

### Survey Design

An online, self-administered survey was developed based on a study in construction [65], and it was deployed using an online survey platform (Qualtrics, Seattle, WA, USA). The survey included a total of 87 items, which were a mix of open-ended and closed-ended questions (see the Appendix). These survey questions were designed to gather information on demographic details, work history, and opinions and perspectives on EXO use in mining. In the present analysis, we did not include responses to questions regarding shift patterns, forms of personal protective equipment, or heat discomfort and associated symptoms. Instead, we only included those items that were relevant to the adoption and use of EXOs.

Before answering the survey questions, participants provided consent and were then required to watch a 5-min introductory video that presented information on arm-support exoskeletons (ASEs), back-support exoskeletons (BSEs), and soft EXOs (Exosuits). Specifically, the video demonstrated how to don and doff EXOs and highlighted the potential benefits of these technologies in mining tasks that involve overhead work and lifting/lowering. The video also provided a verbal description of the user experience, underscoring how EXOs offer physical support to specific body areas. Both the survey and introductory video were made available in English and Indonesian. Survey questions in Indonesian and responses from ID were translated by our two Indonesian investigators (KM and HI), while the introductory video provided captions using the auto-translation feature in YouTube.

### Likelihood of EXO Use, Barriers, and Perceptions

Following the methods used by Gutierrez et al. [65], summation scores were derived by using participant responses to key questions to characterize multiple questions designed to investigate three primary domains: (1) perceived likelihood to use an ASE and BSE in mining (Likelihood to use ASE and BSE scores); (2) perceived concerns or barriers to EXO use in mining (Concerns or Barriers score); (3) perceptions about another worker wearing an EXO (Perceptions score). For questions 4.8 and 4.11 (Appendix) on the respective likelihood to use an ASE and BSE, each response value was obtained on a numerical scale ranging from 0 to 10. For the 11 questions on concerns or barriers to using an EXO (within question 4.21), each of the response choices was assigned a numerical value of: 1 (Yes), 2 (Maybe), and 3 (No). All 11 concerns or barrier responses were summed to have an individual overall concerns or barriers score. For the five questions about perceptions (within question 4.14), response choices on perceptions of EXOs being beneficial, desirable, and cool were provided on a scale from 1 (strongly disagree) to 5 (strongly agree), while the perceptions about colleagues using an EXOs (as injured or weak) was rated on an inverted scale from 1 (strongly agree) to 5 (strongly disagree). Responses were summed for the five perception questions to have an individual overall perception score. Of note, individuals with any missing response (on either barrier, perception, or likelihood to use ASE and BSE) were dropped or excluded when computing the barrier, perception, and likelihood to use scores.

### Data Analysis

Descriptive statistics (i.e., frequency and percentage) were used to summarize demographic and job characteristics for each country. Age and years of experience in mining, originally collected as continuous variables, were categorized into age brackets (18–24, 25–44, 45–64; per Baxter et al., [66]) and experience brackets (0–3, 4–9, 10 + years), respectively. The latter brackets were based on the 25th and 75th percentile values. When relevant, demographic characteristics were compared between the two countries using Fisher’s Exact Test. Differences between countries the age distribution, experience brackets, and years in current job were evaluated using chi-square tests.

To better understand potential differences between the countries, separate analyses of variance (ANOVAs) were performed on barriers, perceptions, likelihood to use ASE, and likelihood to use BSE scores, with *country*, *age bracket*, *experience bracket*, *operation type*, and *mining commodity* as independent variables. We also examined whether the perceived likelihood to use an ASE or a BSE differed between the countries, was associated with demographic or job characteristics, or was influenced by perceptions regarding EXOs. For this, separate Beta regression analyses (i.e., general regression with Beta distribution) were performed on self-reported likelihood values for ASE and BSE. While likelihood values were obtained on a scale of 0 to 10, these values were normalized to a range of 0 to 1 by dividing by 10; such a normalization was done to fit the requirements of the Beta distribution (which is defined on the interval 0–1). Specific independent variables in these analyses were *country*, *age bracket*, *experience bracket*, *operation type*, *mining commodity*, *barriers score*, and *perceptions score*. In our modeling, interaction effects involving *country* were included as predictors for barriers and perceptions scores, since we expected that the relationships between predictors and outcomes might differ between the US and ID.

We also examined miners’ perspectives on EXOs through analysis of their responses to eight open-ended questions in the survey. These questions (see 4.4, 4.5, 4.6, 4.7, 4.10, 4.13, 4.18, 4.19 in the Appendix) focused on the following: (1) concerns regarding or barriers to using EXOs at work; (2) specific tasks where ASEs could be beneficial; (3) specific tasks where BSEs could be beneficial; (4) key features expected of EXOs for use in manual tasks, (5) major reasons for miners to accept and use EXOs at work; (6) body parts that may benefit from EXO use; (7) perceptions of the maximum amount an employer would pay to purchase an EXO; and (8) perception of the maximum amount a miner would pay to purchase an EXO. Responses to each of these questions were first reviewed and then coded separately using an inductive coding method, which involves reading through all responses multiple times to identify initial codes [67–69]. Responses were then grouped into common themes [70–73]. The coding process was iterative, by refining and adjusting themes as new insights emerged. These results are presented separately for the US and ID to highlight differences between the countries. Several direct quotes are provided to capture some important perspectives (these quotes are included verbatim, though corrected for grammar or clarity in some cases). The percentage of responses were compared between the US and ID respondents using *Z*-tests for proportions. All statistical analyses were conducted using JMP Pro V16 (SAS, Cary, NC). Given the exploratory nature of this study, we concluded statistical significance when *p* < 0.1. Summary results are presented as means (SDs).

## Results

A total of 138 respondents completed the survey. Note that three respondents from other countries (i.e., Colombia, Finland, and India) were excluded from the analysis, leaving a total of 135; demographic and job characteristics of the latter are summarized in Table 1. There was not a significant difference (*χ*^*2*^ = 3.06, *p* = 0.22) between experience brackets in the US vs. ID, nor between years in current job (*χ*2 = 0.31, *p* = 0.58). There was a significant difference (*χ*^*2*^ = 24.34, *p* = 0.0001) in the age distribution between the two countries; respondents from the US were older than those from ID, with respective means of 37.7 and 28.2 years. In the job roles category, “Others” included laborers, carpenters, plumbers, pitmen, drivers, plant foremen, welders, and electricians.

**Table 1:** Summary demographics and job characteristics of US and Indonesian (ID) respondents

US	*n* (%)	ID	*n* (%)
**Gender (*n* = 78)**		**Gender (*n* = 57)**	
Male	73 (94)	Male	57 (100)
Female	5 (6)		
**Age (*n* = 78)**		**Age (*n* = 56)**	
18–24 (Young adults)	4 (5)	18–24 (Young adults)	15 (27)
25–44 (Middle-aged)	56 (72)	25–44 (Middle-aged)	40 (72)
45–64 (Older adults)	18 (23)	45–64 (Older adults)	1 (7)
**Years in mining (*n* = 77)**		**Years in mining (*n* = 57)**	
0–3 (Novice)	18 (23)	0–3 (Novice)	20 (35)
4–9 (Experienced)	32 (42)	4–9 (Experienced)	23 (40)
10 + (Highly experienced)	27 (35)	10 + (Highly experienced)	14 (25)
**Years in current job (*n* = 78)**		**Years in current job (*n* = 57)**	
0–3	34 (44)	0–3	26 (46)
4–9	35 (45)	4–9	20 (35)
10 +	9 (11)	10 +	11 (19)
**Job roles (*n* = 76)**		**Job roles (*n* = 57)**	
Operational staff	18 (24)	Operational staff	12 (21)
Technical staff	12 (16)	Technical staff	28 (49)
Management team	5 (6)	Management team	6 (11)
Others	41 (54)	Others	11 (19)
**Mining operations (*n* = 78)**		**Mining operations (*n* = 57)**	
Underground	43 (55)	Surface	54 (95)
Surface	29 (37)	Others	3 (5)
Processing plant	3 (4)		
Others	3 (4)		
**Mining commodities (*n* = 78)**		**Mining commodities (*n* = 57)**	
Stone	34 (44)	Coal	44 (77)
Coal	15 (19)	Metal	11 (19)
Metal	14 (18)	Non-metal	2 (4)
Non-metal	13 (17)		
Sand and gravel	2 (2)		

### Barriers, Perceptions, and Likelihood to Use an ASE and BSE

ANOVA results are presented in Appendix Table 3. Among all predictors, only experience significantly affected barrier scores, which were higher (i.e., fewer perceived barriers) for experienced [24.46 (6.23)] vs. novice [20.55 (6.89)] respondents. Similarly, among all predictors, only work experience significantly affected the likelihood to use BSE scores; these scores were lower for the experienced [7.06 (2.54)] vs. novice [7.24 (2.44)] respondents. Perception scores were higher from US respondents [21.43 (3.47)] compared to those from ID [20.80 (3.26)]. Scores for likelihood to use an ASE differed between age brackets and with the mining commodity. These scores were higher for younger adult respondents [8.37 (2.42)] vs. older adult respondents [6.44 (2.31)], and respondents involved in metal mining operation [8.07 (2.04)] vs. respondents involved in stone mining operation [6.23 (1.98)].

### Predictors of the Likelihood of ASE and BSE Use: Impacts of Demographics, Job Characteristics, and Country

Beta regression results are presented in Table 4 (Appendix). For the likelihood to use an ASE, *age bracket* coefficients were statistically significant, with middle-aged and older respondents indicating they were less likely to use an ASE compared to young respondents. Regarding the *experience bracket*, highly experienced respondents indicated being 34.9% less likely to use an ASE compared to novice respondents. Furthermore, the odds of reporting being likely to use an ASE were 5.1% higher for a unit increase in the barriers score and 12.2% higher for a unit increase in the perceptions score. In our study, a higher score (in the context of concern or barrier) indicates fewer perceived concerns or barriers about using EXOs (accounting for why a higher barrier score was associated with a higher likelihood to use an ASE or BSE).

For a BSE, highly experienced respondents were ~ 49% less likely to indicate being likely to use one compared to novice respondents. Both *barriers score* and the *barriers score* × *country* interaction were also statistically significant predictors. For a unit increase in the barriers score, the odds of indicating being likely to use a BSE increased by 10.5% for US respondents, but only by ~ 3% for ID respondents.

### Opinions and Perceptions Regarding EXO Adoption and Use

#### Concerns About Adopting and Using EXOs

**Fig. 1:**
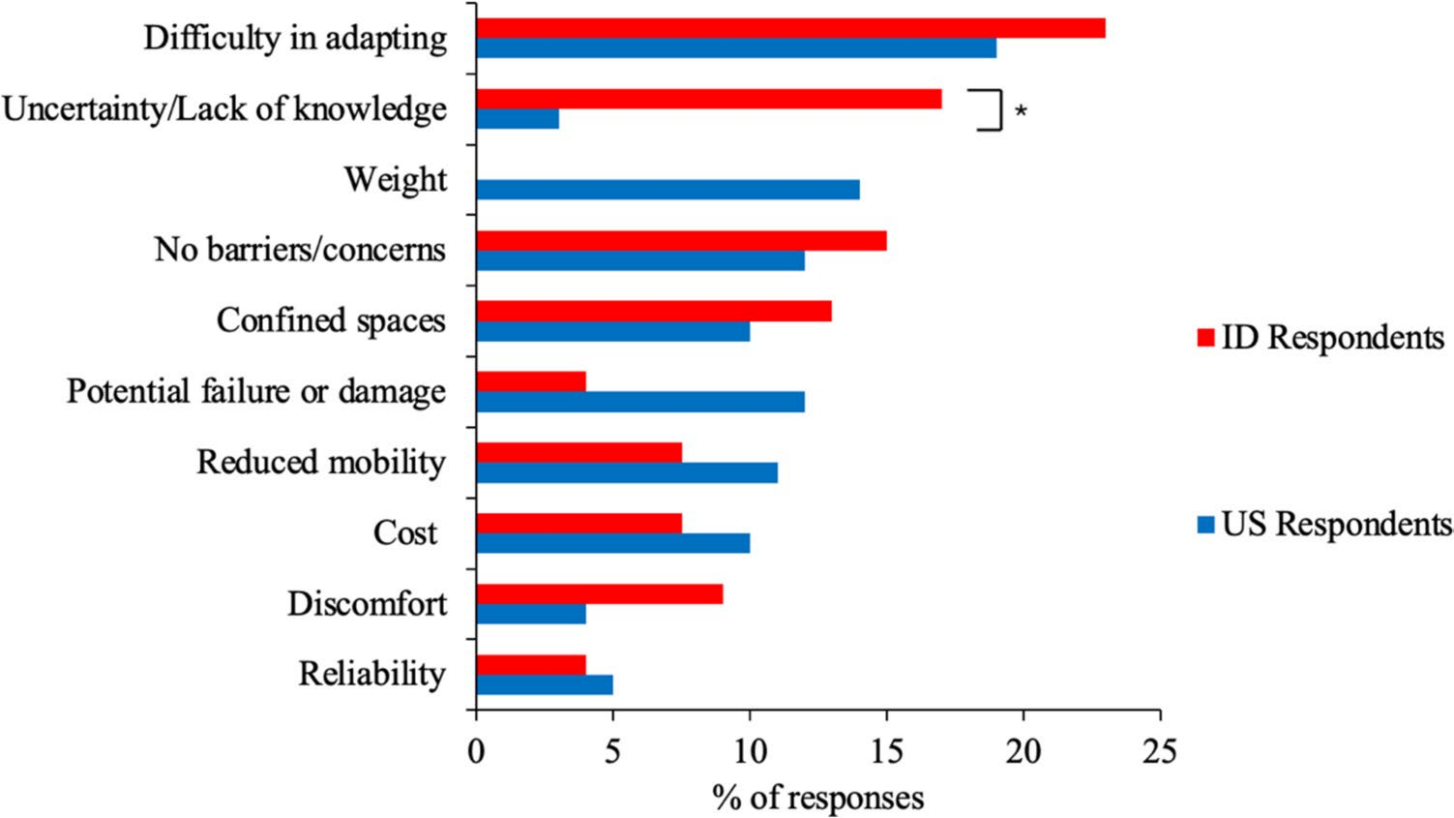
Concerns about adopting and using EXOs. The symbol * denotes a significant difference between countries

Both US and ID respondents reported concerns related to the adoption and use of EXOs, as summarized in Fig. 1 (statistical results are presenting in Table 5 in the Appendix). The most common concern among respondents from both countries was difficulty adapting to EXOs. However, concerns about the weight of EXOs and potential failure or damage were more commonly noted by US respondents. Interestingly, the percentage of respondents indicating no barriers or concerns about adopting and using EXOs was similar in both countries. Overall, the percentage of responses between the two countries were comparable for most concerns, except for uncertainty or lack of knowledge about EXOs, which was significantly larger among ID respondents.

#### Common Health and Safety Hazards

**Fig. 2:**
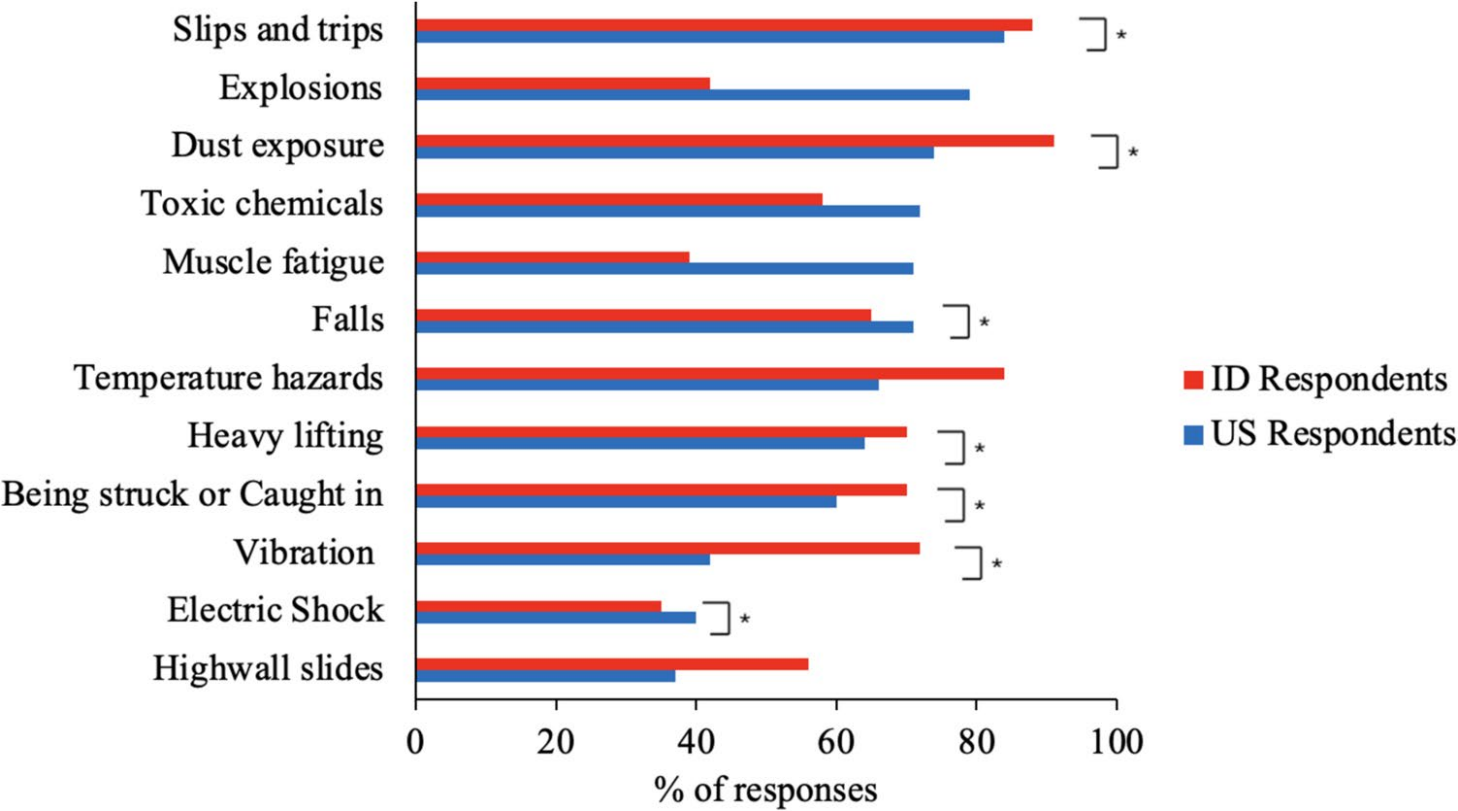
Common health and safety hazards that existed for miners. The symbol * denotes significant differences between countries

The most common perceived health and safety hazard for US respondents was slips and trips, whereas it was dust for ID respondents (Fig. 2). There were several significant differences (Table 5 in the Appendix) between the US and ID respondents. A slightly higher percentage of ID respondents reported hazards related to slips and trips, dust exposure, temperature hazards, heavy lifting, being struck or caught in, vibration, and high-wall slides. A somewhat higher percentage of US respondents reported hazards related to falls and electric shock.

#### EXO Use in the Presence of Common Risks

**Fig. 3:**
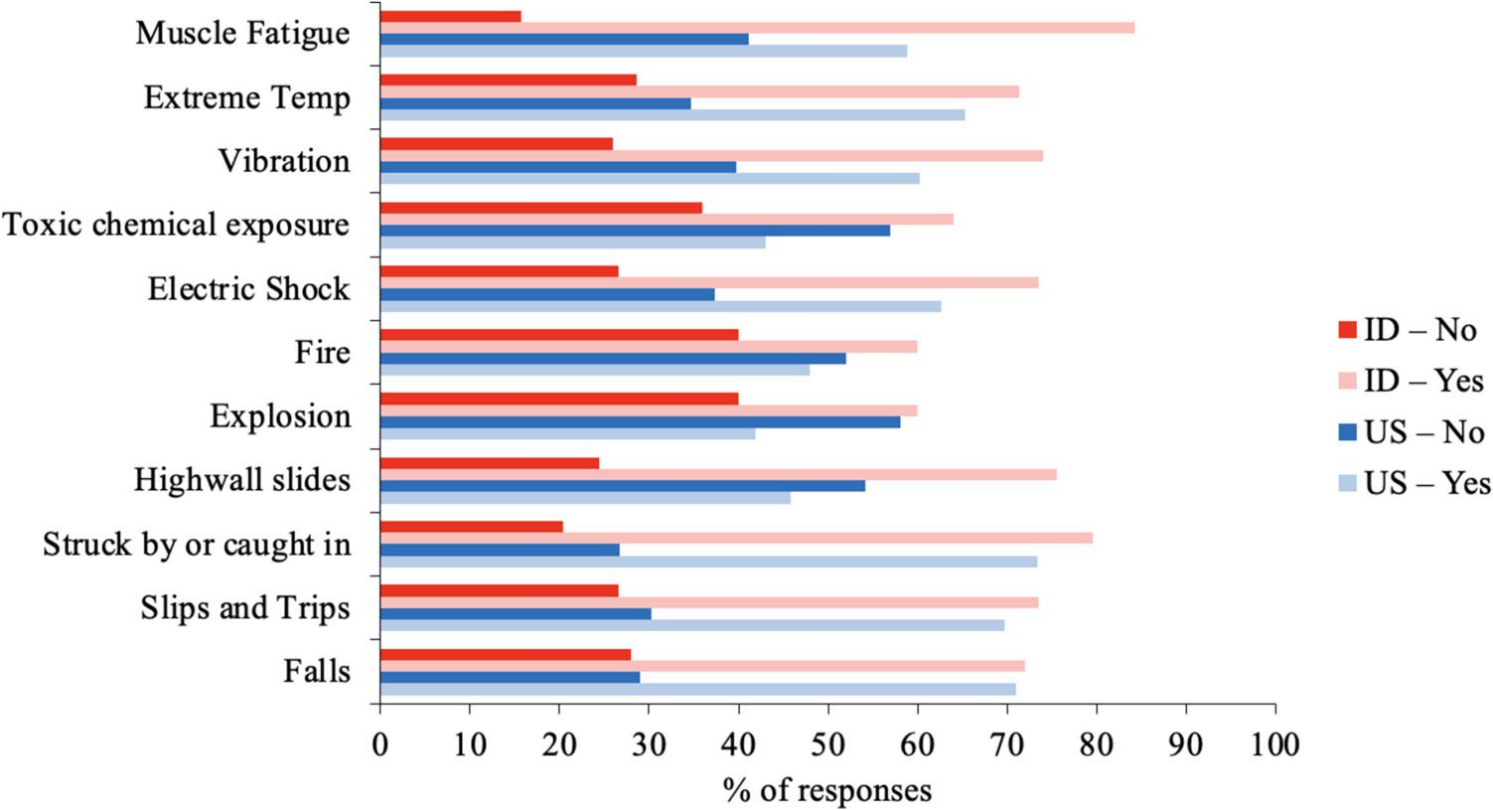
US and ID responses to questions regarding whether they would have concerns using EXOs given specific risks. Note that Yes and Maybe responses were combined into the former category here

ID respondents generally provided more frequent “Yes” responses across most factors (Fig. 3). Interestingly, the percentages of “No” responses were comparable between the two countries for three factors: “Falls,” “Slips and Trips,” and “Struck by or Caught in.” Similarly, the percentages of “Maybe” responses were comparable between the two countries for three factors: “Muscle fatigue,” “Explosion,” and “Highwall slides.” However, US respondents provided more frequent “No” and “Maybe” responses across other factors.

#### Tasks That Could Benefit from ASE or BSE Use

**Fig. 4:**
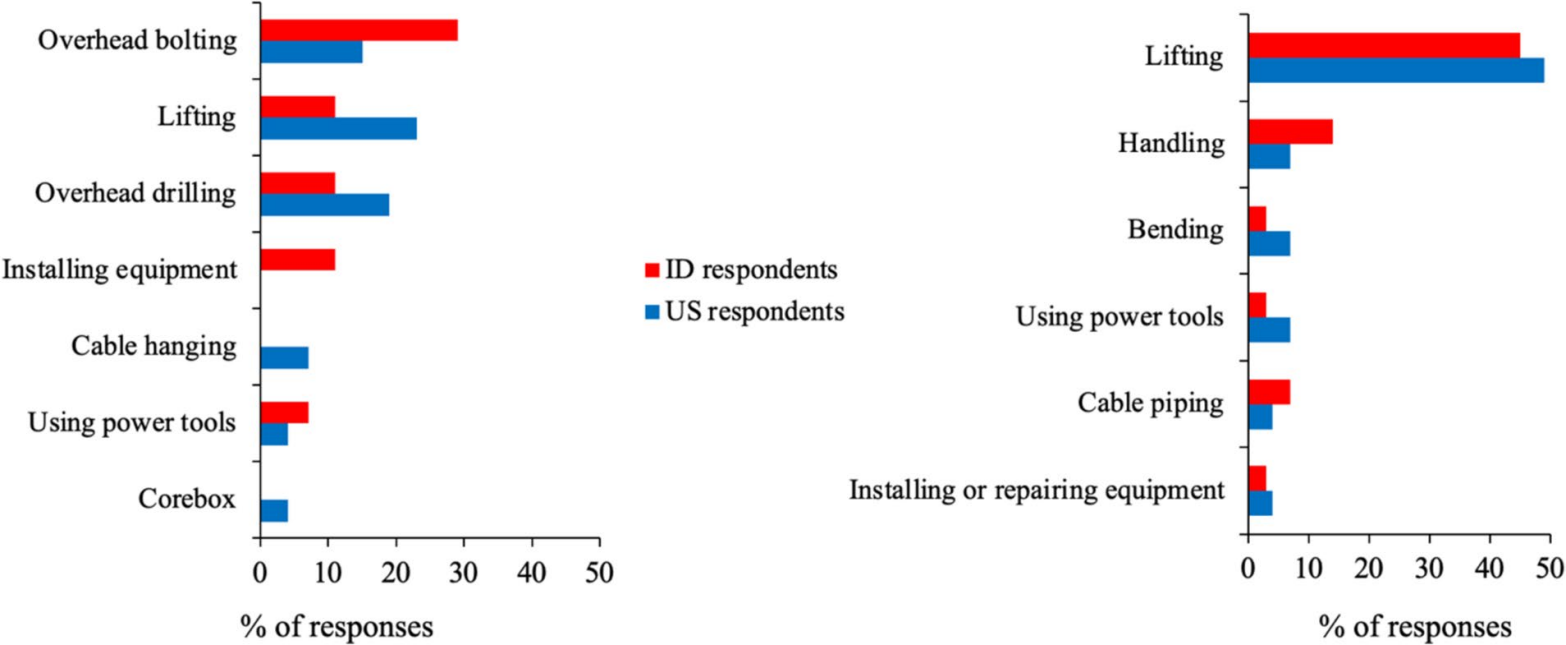
US and ID responses regarding mining tasks that could benefit from using ASEs (left) and BSEs (right)

Several tasks were identified by respondents from both countries, and there were no significant differences in these responses (Fig. 4). Overhead drilling and overhead bolting were the more common tasks reported to benefit from ASE use. Two US respondents expressed that ASEs could be advantageous for any task that requires the arm to be raised for extended periods, noting that *any tasks involving arms raised for several hours*; and *the exoskeleton can reduce fatigue in workers who needs to keep their arms raised for long periods of time while installing or repairing equipment*. Comments from two ID respondents were *support for bolting work*; *Tighten the bolt overhead;* and *Tighten the bolts at the top so that the arms do not get tired easily*.

Interestingly, respondents from both the US and ID indicated lifting as another task that could benefit from ASE use. Though not statistically significant (*p* = 0.20), a higher percentage of US respondents indicated this task (23% vs. 11% for ID respondents). Specific comments made by four US respondents were as follows: *lifting 30–40-lbs pails*; *lifting consistently of heavy materials*; *long arm lift using impact drill work*; and *positions requiring long arm repetitive arm lifting*. A comment made by an ID respondents was that an ASE could help *Lift and hold the weights when installing components*.

In the case of BSE use, the percentage of responses were again generally comparable between the two countries, and lifting was most frequently reported as task that could benefit the most from its use (see Table 5 in the Appendix). Specific scenarios mentioned by five US respondents were as follows: *When bending to reach or lift heavy weights*; *lifting buckets full of sand from one point to another*; *lifting pipe fittings lifting bags of lime*; *lifting drill steel, core boxes, augers, drilling equipment*; and *lifting and carrying electric motors*. Similarly, comments provided by four ID respondents were: *lifting exceeds the reasonable limit, such as lifting toolbox and tire*; *when using a power tool like an impact wrench*; *when loading 24-inch bolts on top of the car*; and *lifting the chain, preparing work equipment*.

#### Features Required for EXO Use in Practice

**Fig. 5:**
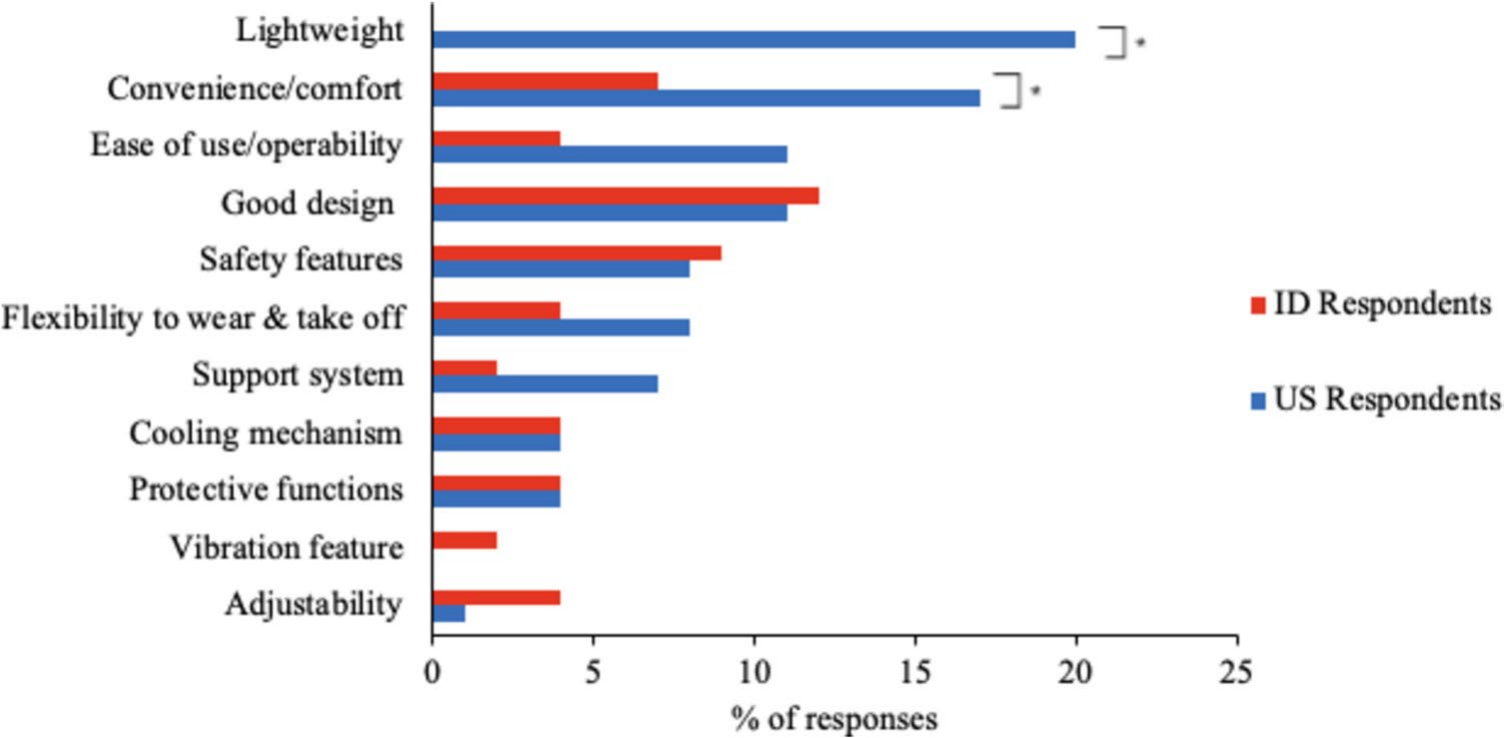
Features required for EXO use in practice. The symbol * denotes significant differences between countries

Among the US respondents, the most important key feature for an EXO was being lightweight, noted by 20% of the respondents (Fig. 5); in contrast, no ID respondents indicated this feature as important, a difference that was statistically significant. Convenience/comfort and ease of use/operability were commonly noted, with higher percentages for the US vs. ID respondents (*p* = 0.1 for the former; difference only approached significance for the latter, with *p* = 0.16).

#### Motivations for Adopting and Using EXOs

Both US and ID respondents reported several key motivations for adopting and using EXOs, including reducing muscle fatigue, decreasing the intensity of work, improving work efficiency, and preventing injuries. Some of the comments from US respondents were: *EXO use would lower fatigue and can help them complete repetitive tasks for extended periods*; *it will reduce workload and makes them feel less tired after work*; *it will reduce the risk of injury and makes work easier*; and *it has the ability to reduce their need for forklifts, jacks, and chain hoists, as well as the ability to lift and hold tools at different positions for a long time*.

#### Acceptable EXO Costs for Employers and Employees

Reported acceptable EXO costs for employers and employees differed between US and ID respondents. About half (49%) of the US respondents reported $US500 as the maximum amount of money a miner would be willing to pay to purchase an EXO, whereas about two-thirds (65%) of the ID respondents reported that less than $US323 (or 5 million IDR) was an acceptable price. However, 50% of the US respondents reported that employers would pay more than $US500 for an EXO, whereas 27% of the ID respondents reported that employers would pay more than $323 (or 5 million IDR) for an EXO. Some of the comments from US respondents were as follows: *If it’s not affordable, no one will decide to buy it. It is tough to estimate a good price as these are really expensive*; and *hard question, but a safety harness runs upwards of $US800 for no smart components. I expect this to be around $US2,000 to 5,000*.

#### Additional Body Parts that May Benefit from EXO Use

For both US and ID respondents, the most frequently reported body part (aside from the shoulders and the back) were the knees (24%) and ankles (12%). This body part was followed by the legs (18%), the arms (10%), and waist/hips (6%) for US respondents; and by the neck (7%), the wrist/hands (5%), and arms (5%) for ID respondents.

#### Union Influence on EXO Adoption

While ID respondents were more frequently unsure (53% vs. 45% for US respondents) whether unions can affect if and how EXOs would be adopted and used in mining, the percentage of responses indicating that unions can influence EXO adoption was comparable between the two countries (35% vs. 33%). Specific comments made by two US respondents were as follows: *If a union pushed for exoskeletons use, it would become more accepted*; and *if the senior employees in a union shop decide it isn’t going to happen, then it doesn’t happen*. On the other hand, a comment made by an ID respondent was: *The union can propose, but the company will follow the existing laws*.

## Discussion

Overall, our study compared the perceptions and concerns of mining personnel in the US and ID regarding adopting EXOs. We specifically examined differences in perceived likelihood of EXO use based on demographics, job characteristics, and EXO perceptions. Our major findings are discussed in detail in the following sections.

### Key Predictors of Miner Perceptions and Likelihood of ASE and BSE Use

We found that while years of experience was the only predictor that affected perceived barriers and the likelihood to use a BSE, country was the only predictor that significantly influenced perception scores. These outcomes suggest that miners with more experience may be more aware of or sensitive to the practical challenges of adopting BSEs, while differences in perception scores between countries may reflect cultural, operational, or contextual factors. However, we found significant differences in the reported likelihood to use ASEs between age groups and across mining commodities. The influence of age may relate to varying physical capabilities or openness to new technologies [74]. Younger workers, for example, may be more apt to accept new technology [75, 76]. Specifically in our study, middle-aged and older respondents, as well as highly experienced individuals, indicated that they would be less likely to use an ASE than younger and less experienced respondents, respectively (See Table 3 and Fig. 3). Although this result aligns with a previous finding in construction [65], it contradicts other studies [59, 77] wherein older workers indicated EXOs as being more helpful than did younger workers. These findings suggest the need to consider age, experience, and contextual factors when promoting the adoption of EXOs.

### Opinions and Perceptions Regarding EXO Adoption and Use

#### Concerns About and Barriers to EXO Use

Both the US and ID respondents expressed concerns about adaptability to EXO use over time (Fig. 1), consistent with previous studies [78–80] that found adaptation to be a key barrier to EXO use. These adaptability concerns may have been influenced by other concerns, such as uncertainties about the reliability of EXOs, ease of use, training requirements, or compatibility of EXOs with work tasks. While most US and ID respondents indicated that they were aware of EXOs, a higher proportion of ID respondents expressed uncertainty or lack of knowledge compared to US respondents (Fig. 1). This uncertainty could be linked to concerns about how well EXOs would integrate into existing workflows and their compatibility with other tools and equipment. One concern raised only by the US respondents was EXO weight (Fig. 1). With 60% of US miners in our study already aware of EXOs, their concerns regarding weight may have reflected a pre-existing understanding of this technology. Indeed, weight is a key factor for EXO adoption [55, 57, 81, 82], as heavy or complex EXOs can affect users’ comfort and range of motion [83].

Confined spaces were noted as a barrier, especially among ID respondents (Fig. 1), which could be due to challenges anticipated with maintaining clearance and the suitability of EXOs in such environments. Schwerha et al. [52] found that workers in manufacturing also reported concerns about EXO rigid components bumping into and damaging products and equipment in confined spaces. They further noted that while some EXO models could be effective in these environments, others may reduce clearance due to the extension of their components.

Another concern noted by both US and ID respondents related to potential failure or damage (Fig. 1). This concern may have been influenced by several factors, including high expectations regarding the reliability and durability of EXOs [52, 58, 73, 84], the nature of mining operations, and the awareness of the financial implications associated with such a technology malfunction or breakdown. US respondents, more than those from ID, indicated concerns about reduced mobility (Fig. 1). Similarly, Nnaji et al. [85] found that workers using EXOs were likely to encounter problems due to the restricted movement that these devices might cause, and Kim and Chung [86] also highlighted mobility as a barrier to EXO use. However, it is unclear why US workers might have found mobility to be a more frequent concern.

In addition to concerns about potential failure or damage, both US and ID respondents also reported some common risk factors in mining as concerns related to EXO adoption (Fig. 3). We also found some differences in the responses between US and ID respondents, with the ID respondents reporting more “Yes” across various factors, compared to US respondents who reported “No” more frequently (Fig. 3). While the exact reasons behind these differences remain unclear, it may be due to the presence of distinct risk factors in their respective workplaces. For example, we would not expect both groups to indicate a willingness to use EXOs in scenarios such as fire, explosion, electric shock, struck by or caught in, and highwall slides. However, it is surprising that most ID respondents expressed concerns about using EXOs given risk factors such as muscle fatigue and vibration, especially since one might expect that their exposure to these hazards (Fig. 2) would increase their willingness to use EXOs as a potential solution. The level of EXO awareness and factors such as differences in safety culture or resource accessibility may also have influenced the responses of both groups regarding concerns about EXO use in the context of these risks.

Furthermore, US and ID respondents had different perceptions about the affordability of purchasing EXOs. The price perspective of the ID respondents about their employers could be due to financial constraints, whereas for the US respondents it may stem from an understanding of the productivity and safety benefits of EXOs. Similar to previous studies from other occupational sectors [34, 52, 55, 65], the cost of purchasing EXOs does appear to be an important potential barrier to miners’ adoption of EXOs. To facilitate EXO adoption in the mining sector, manufacturers may need to address affordability barriers and demonstrate a clear return-on-investment approach.

#### Benefits of EXO Use

Despite the concerns highlighted, both US and ID respondents identified some potential tasks and body parts that could benefit from ASE and BSE use. Though slightly varying in frequency between countries, US and ID respondents identified lifting as a potential task for BSE benefits and overhead tasks for ASE benefits. Although ID respondents worked in surface mines, their perceptions about EXO benefits for overhead bolting tasks may have been influenced by indirect or prior exposures, such as observing others performing such tasks, during training sessions, or from discussions with peers involved in underground operations. Interestingly, US respondents also reported lifting as a task that could benefit from ASEs. These tasks align with typical identified use cases for ASEs and BSEs. Additionally, a higher percentage of US respondents (24%) mentioned the knee as a body part that could benefit from EXOs compared to ID respondents (12%). These variations may be due to differences in mining operations and knowledge about EXOs, reflecting distinct work practices and conditions between the two countries. A majority of both US and ID respondents indicated potential benefits of EXOs related to reducing muscle fatigue and improving work efficiency, and such potential benefits have been measured in some previous studies [52, 56, 57, 65].

#### Promoters or Drivers of EXO USE and adoption

In addition to the preference for a lightweight design, US respondents, more so than ID respondents, emphasized the importance of comfort (Fig. 5). Comfort when wearing an EXO is crucial for EXO adoption [63, 64, 87–90], and discomfort could impact usability [73]. EXOs perceived as comfortable to wear are preferred over those that may cause discomfort [52], and even users who are initially favorably inclined toward EXOs may eventually stop using them if the devices make them feel uncomfortable [74]. Furthermore, comfort is recognized as one of the principal requirements in EXO development, playing a critical role in societal adoption and user acceptance [91, 92]. Considering the physically demanding nature of mining environments, in which miners may be required to wear EXOs for extended periods, any inconvenience or discomfort could result in discontinuation.

Another key feature emphasized by both US and ID respondents is the importance of good design and safety features (Fig. 5). A well-designed EXO should facilitate quick donning and doffing, easy adjustment, and support for the wearer [93–95]. The concerns regarding the convenience and comfort of wearing EXOs might have heightened respondents’ expectations regarding design and safety features possibly due to their unfamiliarity with the technology. It seems worth emphasizing that EXO design should integrate safety components while ensuring they meet the requirements of the end users.

Moreover, US respondents emphasized ease of use more often as a key feature for an EXO to be used in their work (Fig. 5). This feature aligns with the findings of Upasani et al. [58], who identified ease of use as a key factor for EXO adoption in agriculture. The need for easy to use technology may stem from several factors, including the complex nature of mining work and safety concerns. Given that most US respondents in our study were involved in underground mining (Table 1), the complexity associated with their tasks may have led to their preference for an EXO that is simple and intuitive to use. Our results also build on previous findings [96–98], which have similarly shown that perceptions regarding EXO ease of use can influence the intention to use EXOs. Overall, miners may require easy-to-use EXOs that minimize the risk of misuse or any safety concerns.

To supplement our comparisons of overall responses from US and ID mining personnel, we completed an additional analysis to examine whether the results differ when focusing exclusively on US surface mine workers. This was done since most (55%) of our US respondents worked in underground mines whereas 95% of the ID respondents were in surface operations. From this additional analysis, we found that the major trends and conclusions remained consistent with those obtained from the overall US mining cohort, which included both surface and underground mining personnel.

### Limitations

Limitations of the current study should be acknowledged. First, our analysis was of responses from only the US and ID, with unclear representativeness for the broader population of these or other countries. Our study sample had 6% female miners, which is lower than the US national average of 9.4%. While about 6.7% of the mining workforce in ID comprises women, regrettably none of them were able to participate in our study. This underrepresentation may limit the generalizability of our findings regarding any gender-specific barriers, benefits, perceptions, and adoption of EXOs in the mining industry. Our US and ID samples were also unbalanced, in terms of whether respondents worked in surface or underground operations. A second notable limitation of this study is that the respondents from both countries did not have the opportunity to physically experience EXOs during the data collection process. As a result, we are unsure if their responses would have been different if they had used EXOs, which has been shown in some prior work [57, 58]. Future studies could involve conducting in-person or focus group discussions, during which participants would have the opportunity to interact with an exoskeleton.

## Conclusions

Our results highlight notable similarities in responses between US and ID mining personnel regarding the perceived barriers, benefits, and promoters to EXO adoption and use. Both groups recognized the potential benefits of EXOs for tasks such as lifting and overhead work, and shared concerns about adaptation, uncertainty or lack of knowledge, confined spaces, weight, potential failure or damage, and cost. However, there were also some key differences: US respondents indicated they were more likely to use BSEs even though they expressed concerns about their use, while ID respondents, despite reporting more existing health and safety hazards, expressed greater concern about using EXOs given some common risk factors. To facilitate effective adoption, EXOs must be compatible with mining tasks, comfortable, easy to use, and affordable. Training programs, as well as laboratory and field studies, are recommended to identify effective use cases in mining and to address concerns such as safety. Overall, there is strong potential for integrating EXOs into mining operations in both developed and developing countries.

## Supplementary Information

ESM 1 (PDF 368 KB)

## Data Availability

The data obtained during the current study are available from the corresponding author upon reasonable request.
