# Supplementary material for: Perspectives of Mining Personnel on Adopting Occupational Exoskeletons: Comparisons Between a Developed and a Developing Country
Source: Min Metall Explor. 2025 Mar 1;42(2):523–36. doi: 10.1007/s42461-025-01189-1 (PMC11976373; doi:10.1007/s42461-025-01189-1)
Supplement: Supplementary file 1 — (PDF 368 KB) [file 42461_2025_1189_MOESM1_ESM.pdf]

Appendix A

This survey was available in both English and Indonesian.

1. If you would like to participate in this survey, click yes to begin or no to exit knowing that you can decline to answer any question(s) or stop your participation at any time.
   - Yes
   - No

2. Please watch this 5-minute video on exoskeletons and how they are being used in mining.
Did you watch the video?
   - Yes
   - No

2.1 Which country do you work in?
  2.1.1 Which state do you work in?

2.2 Please enter your age

2.3 Please enter your gender

2.4 How long have you worked in mining (years)?

2.5 What is your current job title?

2.6 How long have you worked with your current job (years)?

2.7 Are you a member of any mining worker union(s)?
  2.7.1 Which mining union(s) do you belong to? (Separate multiple entries with commas)

2.8 What is the primary operation at your site?

2.9 What major commodity is mined at your current site?

2.10 What is your typical shift pattern? (e.g., 10 hours per day, 5 days per week)

3.1 Briefly describe the major tasks you need to complete on a typical day, and indicate how much time you generally spend on each task

3.2 Based on your experience during a typical week, how exhausted do you feel at the end of a shift? (Select a response by moving the slider below) - Select an option

3.3 Please describe the equipment that you typically use for your work. For example: power tools such as impact wrenches, or non-powered hand tools such as hammers, shovels, or grease guns.

3.4 What forms of personal protective equipment (PPE) do you use? (Check all that are relevant)
   - Safety glasses
   - Hard hat
   - Face shields
   - Gloves
   - Earplugs

Earmuffs
Reflective vest or other high visibility clothing
Back-support belts
Respirator or mask
Steel-toe shoes or boots with metatarsal protection
Fall protection harness
Other: ___________

3.5 Do you use any of these or other devices that support loads or tools? (Check all that are relevant)
Pallet jack
Tool balancer
Other: ___________
None

3.6 Do you experience discomfort related to heat?
Always
Most of the time
About half of the time
Sometimes
Never

3.6.1 When do you experience discomfort related to heat?
Mainly during hot seasons
On most days

3.7 Which of the following are the reasons for your heat discomfort? (Check all that are relevant)
Working directly under the sun or outdoors
Wearing PPE (personal protective equipment)
Working in small spaces
Hot work (welding, grinding, etc.)
Working near hot sources like driers or kilns in the plant
Other: _____________

3.8 The following asks about body discomfort that you typically experience, including symptoms such as pain, stiffness, spasm, aching, burning, tingling, or numbness. Have you at any time during the last 12 months had symptoms (such as aches, pain, discomfort, numbness) in: Neck, Shoulders, Upper Back, Elbows, Wrists/Hands, Lower back, Hips/Thighs, Knees, Ankles/Feet

During the last 12 months, have you been prevented from carrying out normal activities (e.g., job housework, hobbies) because of these symptoms in: Neck, Shoulders, Upper Back, Elbows, Wrists/Hands, Lower back, Hips/Thighs, Knees, Ankles/Feet
No (1)
Yes (2)

3.9 Please identify common health and safety risks that exist for you or other mining workers? (Check all that are relevant)
Falls (at the same level or from heights)
Slips and Trips
Stuck by or caught in between objects
Heavy lifting
Highwall slides

Explosion
Fire
Electric shock
Toxic chemical exposure
Vibration
Temperature
Muscle fatigue
Dust
Other: _____________

4.1 What is your current knowledge about exoskeletons? Note that we are interested in your level of knowledge about exoskeletons prior to today.
Knowledgeable
Familiar
Heard of it
Never heard of it

4.2 Have you heard about workers using exoskeletons in mining?
Yes
No

4.2.1 What have you heard?
4.2.2 How did you hear about exoskeletons in mining? (Check all that are relevant)
Trainings provided by your employer or co-workers
From a friend or co-worker
Social media (Facebook, Twitter, etc.)
Newspaper
Scientific literature
Online news articles
Podcasts
TV news stations
Magazines
Websites
Youtube videos
Trade shows
Newsletters
Other: _________________
4.2.3 If you have used or are using an exoskeleton, please let us know how long you have used it and which exoskeleton you used.

4.3 What is your first impression based on what you've learned about exoskeletons today? (Select a response by moving the slider below) - Click on a score
0 (Very Negative) to 10 (Very Positive)

4.4 What are some major reasons for you or other mining workers to accept and use an exoskeleton at work?

4.5 What are some major concerns or barriers for you or other mining workers to accept and use an exoskeleton at work?

4.6 What do you think would be important feature(s) that an exoskeleton should have if it is going to be

used during manual mining tasks?

4.7 Considering all the different tasks that you or other mining workers need to complete, what are some specific tasks that you think an arm-support exoskeleton may be helpful for? (Enter new tasks on a different line; Please leave this part blank if you have nothing add.)

4.7.1 How is the quality of such tasks measured? (e.g., visual inspection, specific task performance metrics, etc.)

4.8 If available at work now, how likely are you to use an arm-support exoskeleton? (Select a response by moving the slider below) - Click on a number

0 to 10

4.9 Please explain why you responded this way.

4.10 Considering all the different tasks that you or your workers need to complete, what are some tasks that you think a back-support exoskeleton may be helpful for? (Enter new tasks on a different line; Please leave this part blank if you have nothing add.)

4.10.1 How is the quality of such tasks measured? (e.g., visual inspection, specific task performance metrics, etc.)

4.11 If available at work now, how likely are you to use a back-support exoskeleton? (Select a response by moving the slider below) - Click on a number

0 to 10

4.12 Please explain why you responded this way.

4.13 For manual mining tasks, can you think of any other body parts that may benefit from an exoskeleton, other than the shoulders and the back?

4.13.1 Why do you think these body parts would benefit from using an exoskeleton?

4.14 If you saw someone wearing an exoskeleton, how strongly would you agree or disagree with the responses listed below?

The exoskeleton is helping them
I want one as well
The worker must be physically weak
How cool!
The worker must be injured

4.15 What do you think is the maximum amount of time it should take to put on an exoskeleton? For example, less than 30 seconds, 1-5 minutes, etc.

4.16 What do you think is the maximum amount of time it should take to remove an exoskeleton?

4.17 Do you think an exoskeleton is:

Personal protective equipment (PPE, like a hard hat or steel-toed shoes)
A work tool
Other: _________________

4.18 What do you think is the most amount of money an employer would be willing to pay to purchase an exoskeleton?

4.19 What do you think is the most amount of money a mine worker would be willing to pay to purchase an exoskeleton?

4.20 Based on your understanding, do you think training and education for using an exoskeleton should be provided?

4.20.1 Please explain why you think so

4.21 Would you have concerns using a back-support or an arm-support exoskeleton given these risks? –

Falls (at the same level or from heights)
Slips and trips, Struck
Struck-by or caught in-between objects
Highwall slides
Explosion
Fire
Electric shock
Toxic chemical exposure
Vibration
Extreme temperature
Muscle fatigue

4.22 Do you think unionization might affect if and how exoskeletons would be adopted and used in mining?

4.22.1 Please explain why you think so

4.23 Is there anything else you would like to comment on about exoskeletons?

Appendix B

Table A1 Raw Perception, Likelihood to use ASE and BSE, and Concerns or Barriers Score Calculations.

Perception Score Calculation

Survey Question	Participant response	Assigned Value
(1) The exoskeleton is helpful	Strongly agree	5
	Somewhat agree	4
	Neither agree nor disagree	3
	Somewhat disagree	2
	Strongly disagree	1
(2) I want one as well	Strongly agree	5
	Somewhat agree	4
	Neither agree nor disagree	3
	Somewhat disagree	2
	Strongly disagree	1
(3) The worker must be physically weak	Strongly agree	1
	Somewhat agree	2
	Neither agree nor disagree	3
	Somewhat disagree	4
	Strongly disagree	5
(4) How cool	Strongly agree	5
	Somewhat agree	4
	Neither agree nor disagree	3
	Somewhat disagree	2
	Strongly disagree	1
(5) The worker must be injured	Strongly agree	1
	Somewhat agree	2
	Neither agree nor disagree	3
	Somewhat disagree	4
	Strongly disagree	5

Perceived Likelihood to Use Score Calculation		
**Survey Question**	**Participant's response**	**Assigned Value**
(1) likelihood to use ASE	0	0
	1	1
	2	2
	3	3
	4	4
	5	5
	6	6
	7	7
	8	8
	9	9
	10	10
(2) likelihood to use BSE	0	0
	1	1
	2	2
	3	3
	4	4
	5	5
	6	6
	7	7
	8	8
	9	9
	10	10

Concerns or Barriers Score

Survey Question	Participant response	Assigned value
(1) Fall	No	3
	Maybe	2
	Yes	1
(2) Slips and Trips	No	3
	Maybe	2
	Yes	1
(3) Struck by or caught in	No	3
	Maybe	2

	Yes	1
(4) Highwall slides	No	3
	Maybe	2
	Yes	1
(5) Explosion	No	3
	Maybe	2
	Yes	1
(6) Highwall slides	No	3
	Maybe	2
	Yes	1
(7) Electric Shock	No	3
	Maybe	2
	Yes	1
(8) Chemical Exposure	No	3
	Maybe	2
	Yes	1
(9) Vibration	No	3
	Maybe	2
	Yes	1
(10) Extreme temperature	No	3
	Maybe	2
	Yes	1
(11) Muscle Fatigue	No	3
	Maybe	2
	Yes	1

Table A2 ANOVA Results for Barriers, Perceptions, Likelihood to use ASE and BSE scores by country, age brackets, experience brackets, mining commodity, and operation type (bold indicates *p*-value <0.1)

		**Barriers**				**Perceptions**				**Likelihood to use ASE**				**Likelihood to use BSE**			
**Source**	**Level**	***n***	**Mean**	**SD**	***F*(*p*)**	***n***	**Mean**	**SD**	***F*(*p*)**	***n***	**Mean**	**SD**	***F*(*p*)**	***n***	**Mean**	**SD**	***F*(*p*)**
Country	INDS	46	19.83	7.6	1.08 (0.302)	50	20.8	3.26	3.44 **(0.067)**	56	7.64	2.61	0.20 (0.655)	52	7.44	2.59	0.36 (0.553)
	U.S.	72	23.68	5.56		70	21.43	3.47		72	6.94	2.32		74	7.22	2.22	
Age bracket	Middle-aged	84	21.36	6.59	1.25 (0.292)	85	21.64	3.15	1.15 (0.322)	90	7.20	2.45	2.8 (**0.065**)	89	7.40	2.29	0.38 (0.685)
	Older adults	17	26.06	4.64		16	19.75	3		18	6.44	2.31		17	7	2.12	
	Young adults	16	22.06	7.97		18	20.33	4.39		19	8.37	2.42		19	7.53	2.48	
Experience bracket	Highly Exp	37	24.46	6.23	2.46 (**0.09**)	38	21.21	3.18	0.12 (0.886)	39	7.28	2.15	1.24 (0.295)	39	7.72	2.06	2.45 (**0.091**)
	Exp	48	21.54	6.55		48	21.42	3.85		53	7.24	2.57		53	7.06	2.54	
	Novice	33	20.55	6.89		34	20.76	2.94		36	7.24	2.69		34	7.24	2.44	
Major commodity	Coal	52	20.25	6.79	0.19 (0.94)	51	21.12	3.40	1.17 (0.33)	57	7.68	2.50	2.66 (**0.037**)	53	7.58	2.32	1.41 (0.234)
	Metal	20	22.25	7.25		23	21.87	2.75		24	8.07	2.04		25	7.88	2.40	
	Non-metal	14	23.64	7.42		14	19.86	5.13		14	6.33	3.18		15	6.33	2.72	
	Sand & Gravel	2	25.50	0.71		2	23.00	2.83		2	9.00	1.41		2	9.00	1.41	
	Stone	30	24.33	5.28		30	21.27	2.82		31	6.23	1.98		31	7.00	2.22	
Operation type	Undergrou nd	40	24.28	5.24	1.03 (0.381)	37	20.68	3.96	1.63 (0.187)	40	7.18	2.37	0.19 (0.90)	40	7.38	2.29	0.93 (0.428)
	Surface	71	21.11	7.26		76	21.29	3.19		81	7.30	2.56		77	7.53	2.38	
	Processing plant	2	16.50	3.54		3	23.67	1.15		2	7.00	1.41		3	5.67	2.52	
	Others	5	21.60	6.19		4	22.00	1.15		5	7.60	2.51		6	6.17	2.79	

Table A3 Beta regression results for the perceived likelihood of ASE and BSE use.

**Parameter**	ASE				BSE			
	**Coef.**	**S.E.**	***p* value**	**95% CI**	**Coef.**	**S.E.**	***p* value**	**95% CI**
Intercept	-2.02	1.02	**0.05**	-4.01, -0.02	-1.73	0.98	0.08	-3.6, 0.20
Country[INDS - U.S.]	0.25	0.48	0.61	-0.69, 1.19	-0.10	0.48	0.84	-1.03, 0.83
Age[Middle - Young]	-0.70	0.34	**0.04**	-1.37, -0.02	-0.13	0.34	0.70	-0.79, 0.53
Age[Old - Young]	-1.32	0.52	**0.01**	-2.34, -0.29	-0.28	0.50	0.58	-1.26, 0.70
Experience[Exp. - Novice]	-0.07	0.35	0.84	-0.75, 0.61	-0.40	0.35	0.25	-1.08, 0.28
Experience[Highly Exp. - Novice]	-0.43	0.31	**0.17**	-1.03, 0.18	-0.68	0.32	**0.03**	-1.30, -0.06
Operation[Other - Underground]	0.30	0.76	0.69	-1.18, 1.78	-0.30	0.66	0.66	-1.60, 1.00
Operation[Surface - Underground]	-0.16	0.39	0.68	-0.94, 0.61	0.48	0.39	0.22	-0.29, 1.25
Commodity[Coal - Stone]	0.44	0.46	0.34	-0.46, 1.34	0.16	0.45	0.73	-0.72, 1.04
Commodity[Metal - Stone]	0.75	0.45	0.10	-0.14, 1.64	0.48	0.45	0.29	-0.41, 1.37
Commodity[Non-metal - Stone]	0.08	0.42	0.84	-0.74, 0.90	-0.68	0.40	**0.08**	-1.46, 0.09
Barriers score	0.05	0.03	**0.06**	0.00, 0.11	0.10	0.03	**0.00**	0.04, 0.15
Perceptions score	0.11	0.05	**0.01**	0.02, 0.20	0.05	0.04	0.26	-0.04, 0.13
Barriers score×Country[INDS - U.S.]	-0.05	0.04	0.14	-0.12, 0.02	-0.07	0.04	**0.06**	-0.14, 0.00
Perceptions score×Country[INDS - U.S.]	-0.09	0.07	0.18	-0.23, 0.04	0.01	0.07	0.86	-0.12, 0.15

Table A4 *Z*-test results for the percentage of miner responses on opinions and perspectives on EXOs.

Category	Themes	U.S. (% of responses)	INDS (% of responses)	***Z*-statistic**	***P*-value**
**Concern about adopting and using EXOs**	Difficulty in adapting	19	23	-0.542	0.59
	Uncertainty /lack of knowledge	3	17	-2.682	**0.007**
	Weight	14	0	2.839	0.005
	No barrier/concerns	12	15	-0.485	0.628
	Confined spaces	10	13	-0.5201	0.603
	Potential failure/damage	12	4	1.571	0.116
	Reduced mobility	11	7.5	0.656	0.512
	Cost	10	7.5	0.482	0.63
	Discomfort	4	9	-1.143	0.253
	Reliability	5	4	0.263	0.793
**Potential task that could benefit from ASE**	Overhead bolting	15	29	-1.446	0.148
	Lifting	23	11	1.277	0.202
	Overhead drilling	19	11	0.902	0.367
	Installing	0	11	-2.321	0.02
	Cable hanging	7	0	1.406	0.16
	Using power tools	4	7	-0.565	0.572
	Corebox	4	0	1.053	0.293
**Potential task that could benefit from BSE**	Lifting	49	45	0.333	0.739
	Handling	7	14	-0.983	0.325
	Bending	7	3	0.731	0.465
	Using power tools	7	3	0.731	0.465
	Cable piping	4	7	-0.564	0.573
	Installing or repairing	4	3	0.223	0.824
**Key features required for EXO use in practice**	Lightweight	20	0	3.429	0.0006
	Convenience or comfort	17	7	1.641	0.101
	Ease of use/operability	11	4	1.410	0.158
	Good design	11	12	-0.172	0.863
	Safety features	8	9	-0.197	0.844
	Flexibility to wear and take off	8	4	0.901	0.367
	Support system	7	2	1.271	0.204

	Cooling mechanism	4	4	0	1
	Protective function	4	4	0	1
	Vibration feature	0	2	-1.197	0.231
	Adjustability	1	4	-1.104	0.27
**Common health and safety hazard**	Slips and trips	37	56	-2.179	**0.03**
	Explosions	40	35	0.588	0.556
	Dust exposure	42	72	-3.441	**0.0006**
	Toxic chemicals	60	70	-1.191	0.234
	Muscle fatigue	64	70	-0.726	0.468
	Falls	66	84	-2.334	**0.02**
	Temperature hazards	71	65	0.737	0.461
	Heavy lifting	71	39	3.692	**0.0002**
	Being struck or caught in	72	58	1.687	**0.092**
	Vibration	74	91	-2.488	**0.013**
	Electric shock	79	42	4.377	**0.00001**
	Highwall slides	84	88	-0.652	0.514
